# A Rhizosphere-Derived Consortium of *Bacillus subtilis* and *Trichoderma harzianum* Suppresses Common Scab of Potato and Increases Yield

**DOI:** 10.1016/j.csbj.2019.05.003

**Published:** 2019-05-15

**Authors:** Zhenshuo Wang, Yan Li, Lubo Zhuang, Yue Yu, Jia Liu, Lixia Zhang, Zhenjiang Gao, Yufeng Wu, Wa Gao, Guo-chun Ding, Qi Wang

**Affiliations:** a Department of Plant Pathology, MOA Key Lab of Pest Monitoring and Green Management, College of Plant Protection, China Agricultural University, Beijing 100193, China; b Chongqing Key Laboratory of Economic Plant Biotechnology, Collaborative Innovation Center of Special Plant Industry in Chongqing, Institute of Special Plants, Chongqing University of Arts and Sciences, Yongchuan, Chongqing 402160, China; c Baotou Academy of Agricultural and Animal Husbandry Sciences, Baotou 014000, Inner Mongolia, China; d Agricultural Technology Extension Center of Baotou City, Baotou 014000, Inner Mongolia, China; e College of Resources and Environmental Science, Beijing Key Laboratory of Biodiversity and Organic Farming, China Agricultural University, Beijing 100193, China; f College of Forestry and Life Science, Chongqing University of Arts and Sciences, Yongchuan, Chongqing 402160, China

**Keywords:** Potato common scab, Microbial product, Rhizosphere bacterial communities, *Bacillus subtilis*, *Trichoderma harzianum*

## Abstract

The ability of a rhizosphere-derived microbial product (composed of a consortium of a strain of *Bacillus subtilis* and a strain of *Trichoderma harzianum*) to suppress common scab disease in potato caused by *Streptomyces* spp. was examined over a two-year period. Relative to the condition in which 0 kg·ha^−1^ of the designated microbial product was applied (control), the disease index decreased by 30.6%–46.1%, and yield increased by 23.0%–32.2% in treatments in which 225 or 300 kg·ha^−1^ of the microbial product was administered, respectively. The bacterial communities present in the rhizosphere were assessed at an early stage of tuber formation, a time at which tubers are susceptible to common scab. Potato plants in which soils were treated with 225 or 300 kg·ha^−1^ of the microbial product harbored rhizospheric microbiota with lower α-diversity and an increased relative abundance of taxa representing the beneficial bacteria. In summary, a select microbial product composed of a consortium of *Bacillus subtilis* and *Trichoderma harzianum* effectively suppressed common scab disease and increased tuber yield by establishing a high relative abundance of beneficial bacteria in the rhizosphere.

## Highlights

• Microbial product effectively suppressed potato common scab.
• Microbial product markedly increased potato yield.
• Microbial product recruited beneficial bacteria in the potato rhizosphere.

## Graphical Abstract

**Figure:**
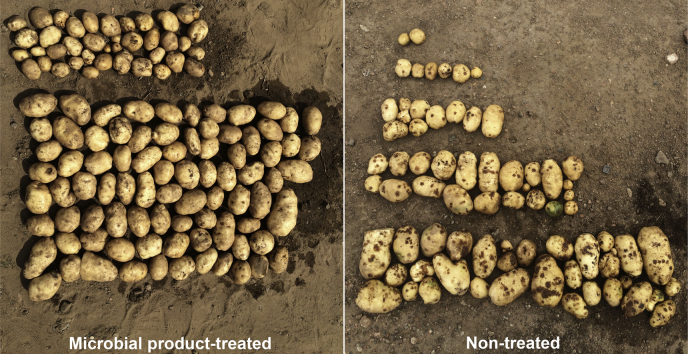

## 1 Introduction

Potato (*Solanum tuberosum* L.) ranks as the fourth main food crop produced after rice, wheat, and maize [1]. Potato common scab (PCS) disease is a recurrent soil-borne disease, mainly caused by *Streptomyces* spp. [2], that is responsible for significant economic losses [3]. Various control measures, such as the use of agrochemicals, organic amendments, crop rotation, and the use of microbial bioagents have been utilized to manage this disease [4]. Among these methods, the use of bioagents is of great interest due to their low environmental impact [5]. Members of *Bacillus* spp., *Pseudomonas* spp., and *Trichoderma* spp. have been demonstrated to suppress several soil-borne pathogens, including *Streptomyces* spp. and *Fusarium* spp. and have also been shown to promote plant growth [4,6,7]. Thus, they can serve simultaneously as both a biopesticide and a biofertilizer. Successful control of soil-borne diseases, including PCS, by bioagents has been reported [5,8]. The impact of the bioagents, however, has been variable, especially under field conditions [5]. Despite several hypotheses regarding the mechanism of these bioagents, such as pathogen antagonism, niche competence, and acting as keystone taxa, the actual mechanisms associated with successful suppression of PCS and other plant diseases by bioagents remain unclear.

The importance of the composition of the rhizosphere microbiome on plant health and productivity has been increasingly recognized. Some studies have indicated that the application of bioagents, belonging to *Bacillus* spp. or *Trichoderma* spp., suppresses soilborne plant diseases and alters the composition of the rhizosphere microbial community in banana, cucumber, and potato [[9], [10], [11]]. It is still unknown, however, whether shifts in the composition of rhizospheric microbial communities is a prerequisite for the successful biological control of PCS. In addition to the application of bioagents, several other factors can also influence the composition of rhizosphere microbiota, including plant development, plant species, soil physico-chemical properties, and organic amendments [[12], [13], [14]]. These additional factors often make comparisons between different studies problematic.

Previously, a microbial product composed of a consortium of *Bacillus subtilis* (strain znjdf1) and *Trichoderma harzianum* (strain znlkhc1) was tested in a potato field trial in 2015 in Inner Mongolia, China. In that field trial, the average PCS disease index decreased by 28% and 41% and total tuber yields increased 21.8% and 31.5% in two different field sites, respectively, that were treated with 300 kg·ha^−1^ of the microbial product. We hypothesized that the microbial product, composed of a consortium of *Bacillus subtilis* and *Trichoderma harzianum,* shifts the composition of the microbial community of the potato rhizosphere during the early stages of tuberization, a critical stage for PCS development [[15], [16], [17], [18]]. In the present study, a two-year (2016–2017) field study was conducted using the same microbial product at four levels of application 0, 150, 225, and 300 kg·ha^−1^. Bacterial diversity in the potato rhizosphere was assessed by high throughput amplicon sequencing of the 16S rRNA gene to determine the effect of the microbial product on the α-diversity and β-diversity of bacteria in the rhizosphere microbiome, and to identify the key taxa associated with the suppression of PCS. The results of the study provide information on the potential mechanism of the ability of a commercial microbial consortium to improve the health and productivity of potato.

## 2 Material and Methods

### 2.1 Experimental Design and Application of the Microbial Product

A two-year (2016–2017) field study was conducted in Guyang County (40^o^58′ N, 109^o^53′ E), Baotou City, Inner Mongolia, China. The treatments used in the study included the application of four different levels of a microbial product, 0 (CK), 150 (B150), 225 (B225), or 300 (B300) kg·ha^−1^ utilizing a randomized complete block design. Each treatment consisted of three replicated blocks, with each block measuring 23 m × 24 m. The soil type was a sandy soil. The field had been used for potato production for six years and plantings suffered from a high level of potato common scab in 2015.

The commercial microbial product, Micro-ecological Agents for Controlling Continuous Crop Diseases (Sino Green Agri-Biotech Co. Ltd., Beijing, China) was used in the experiment. The microbial product is composed of a consortium of *Bacillus subtilis* strain znjdf1 (strain accession number: CGMCC NO.7850) and *Trichoderma harzianum* strain znlkhc1 (strain accession number: CGMCC NO.7861) and an inert carrier (diatomaceous earth). The concentration of *B. subtilis* strain znjdf1 and *T. harzianum* strain znlkhc1 in the final preparation of the microbial product were 9.5 × 10^8^ CFU·g^−1^ and 0.5 × 10^8^ CFU·g^−1^, respectively, according to the product description. Planting furrows were made in the selected filed sites and ‘Kexin No. 1’ potato seed tubers were placed in the furrows. Different concentrations of a microbial product (0, 150, 225, 300 kg·ha^−1^) were then added to the furrows. The microbial product was applied in the furrows around the potato seed tubers. The only difference between control and treatments was the amount of microbial product used. All fields, regardless of treatment, were subjected to the same local system of agricultural management used for growing potatoes, which did not utilize the application of any agrochemicals, such as pesticides, herbicides, or chemical fertilizers.

Bulk soil samples were collected three days prior to planting and rhizosphere samples were collected at the time of early tuber formation. Briefly, the root system of a collected plant was first vigorously shaken to detach loosely adhering soil. The thin layer of rhizosphere soil was then carefully separated and collected using a small brush and placed in a sterile plastic bag. Each replicate consisted of the pooled rhizosphere soil obtained from 15 plants. All samples were kept on ice during transport to the laboratory. Samples were stored at −80 °C prior to the extraction of DNA.

### 2.2 Determination of Potato Common Scab (PCS) Disease Index and Potato Yield

A PCS disease index was calculated at harvest. The degree of tuber disease severity was rated as follows: 0 = no lesion; 1 = scattered lesions covering <25% of the tuber surface; 2 = extensive lesion covering >25% but <50% of the tuber surface; 3 = extensive lesion covering >50% but <75% of the tuber surface, and 4 = extensive lesions covering >75% of the tuber surface. The disease index for each treatment was calculated using the following formula:

$$\text{Disease index}=\left({\Sigma_{i=0}}^{4}N_{i}\times i\right)/\left(4\times {\Sigma_{i=0}}^{4}N_{i}\right)\times 100\%$$

Where *i* is the severity (0–4), 4 is the highest level, and *N*_*i*_ is the number of tubers with a severity of *i*. For yield assessment, all potatoes in each treatment plot were manually harvested and weighed.

### 2.3 DNA Extraction From Soil Samples

Total DNA was extracted from 0.5 g of a soil sample using the MP DNA Isolation Kit (MO BIO Laboratories, Carlsbad, CA, USA) according to the manufacturer's instructions. The concentration and quality (A260/A280) of the DNA were assessed by agarose gel electrophoresis and a spectrophotometer (NanoDrop ND-2000, Wilmington, DE, USA).

### 2.4 PCR Amplification, Sequencing, and Sequence Analysis

The bacterial 16S rRNA gene was amplified using the universal primers 515F (5′-GTGCCAGCMGCCGCGGTAA-3′) and 909R (5′- CCCCGYCAATTCMTTTRAGT -3′) along with a 12 nt unique barcode for each sample. Three individual reactions were conducted on each sample to mitigate potentially heterogeneous amplification from the environmental templates. The PCR products were purified using a QIAquick Gel Extraction Kit (QIAGEN, Germany), and quantified using quantitative real-time PCR. The products from all three amplicon libraries were mixed at equal moles for deep sequencing on an Illumina Mi-seq system using the Reagent Kit v2 2 × 250 bp as described in the manufacture manual.

The 16S rRNA sequence data was subjected to quality filtering so that only high-quality sequences (length > 300 bp, without ambiguous base ‘N’, and average base quality score > 20) were used in the downstream analyses. Primer and barcode regions were removed along with chimeric sequences using ChimeraSlayer software. The remaining sequences were than subjected to a standalone BLASTN analysis against the SILVA database [19]. Sequences were categorized into operational taxonomic units (OTUs) using an identity of 97% [20]. The Ribosomal Database Project (RDP) Classifier tool (Classifier, version 2.6) was used to assign sequences into different taxonomic groups at a confidence threshold of 50% [21]. Diversity index including Chao1 and Shannon were calculated by 100 times re-sampling of an equal number of sequences from each sample using the ‘vegan’ package. Taxa with different relative abundance were identified as previously described [19]. β-diversity of the rhizospheric samples was analyzed using principle component analysis (PCA) based on the Bray-Curtis dissimilarity index [22,23]. To explore the correlation between yield or disease severity and the composition of the rhizosphere microbial community, redundancy analysis was performed using a forward selection to avoid using collinear parameters [19]. To identify taxa with a significant difference in relative abundance between treatments, multiple comparisons were performed using a negative binomial model. All statistical analyses were carried out with using R 3.1.2 (http://www.r-project.org/) software. All tools were incorporated into the galaxy instance (http://www.freebioinfo.org) according to the description of the galaxy development team (https://galaxyproject.org/). The raw sequences obtained from the analysis of this study are available in the NCBI's Sequence Read Archive (SRA) repository as SRP175095.

## 3 Results and Discussion

### 3.1 High Doses of the Microbial Product Reduced the Disease Index for Potato Common Scab (PCS) and Increased Potato Tuber Yield

**Fig. 1:**
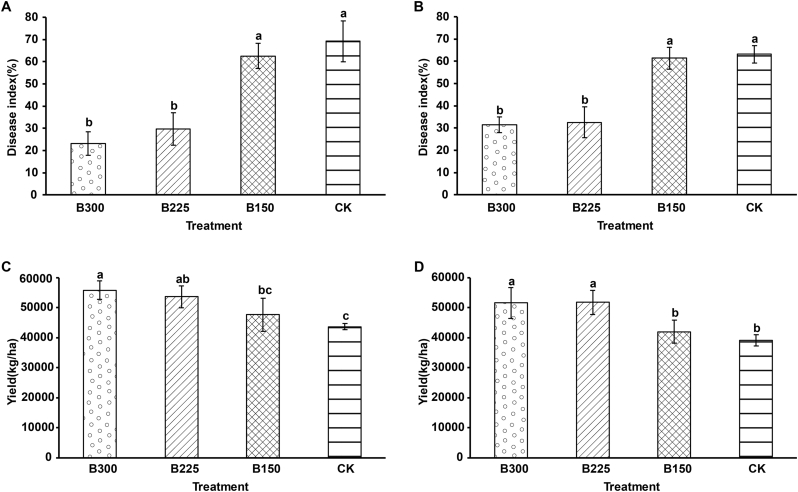
Disease index of potato common scab (A, 2016; B, 2017) and tuber yield (C, 2016; D, 2017) in plots treated with 0 (CK), 150 (B150), 225 (B225), and 300 (B300) kg·ha^−1^ of a microbial product, composed of a consortium of *Bacillus subtilis* and *Trichoderma harzianum*. Data represent the mean ± standard deviation (n = 3). Significant differences between treatments and the control were determined by ANOVA. Significantly different means (p < 0.05) are indicated by different letters above each bar.

PCS disease severity was significantly lower in both 2016 and 2017 in plantings that received high doses (225 kg·ha^−1^ and 300 kg·ha^−1^) of the microbial product than in plantings that received no application (CK) of the microbial consortium. In 2016, common scab symptoms were observed on tubers in all of the treatments. The average disease index for the B150, B225, and B300 treatments was 62.6%, 29.6%, and 23.1%, respectively (Fig. 1A), while the average disease index in the CK treatment plots was approximately 69.2%. Similar results were obtained in 2017, with the average disease index in the CK, B150, B225, and B300 treatment plots being 63.2%, 61.4%, 32.6%, and 31.5%, respectively (Fig. 1B). In 2016, potato yields in the B150, B225, and B300 treatment plots were 47,741, 53,661 and 55,877 kg·ha^−1^, respectively, while yield in the CK plot was only 43,642 kg·ha^−1^ (Fig. 1C). Similar yields in the various treatment plots were obtained in 2017 (Fig. 1D). Yields in the B150, B225, and B300 treatment plots were 42,025, 51,784, and 51,611 kg·ha^−1^, respectively, while yield in the CK plot was only 39,179 kg/ha^−1^ (Fig. 1D).

In the present study, potatoes had been continually cropped in the selected fields for six years and the PCS disease severity in the CK plots had reached 69.2% in 2016 and 63.2% in 2017. This level of severity is in agreement with a previous study by Larkin et al. [24] where the monoculture of potato frequently led to an increase in PCS and a significant decline in yield. Our results indicate, however, that the application of the microbial product at 225 kg·ha^−1^ and 300 kg·ha^−1^ greatly suppressed the PCS disease index and increased potato tuber yield, relative to untreated plots. The reduction in the PCS disease index (46.1% and 31.7% in 2016 and 2017, respectively) obtained by the application of 300 kg·ha^−1^ of the microbial product, composed of a consortium of *Bacillus subtilis* and *Trichoderma harzianum,* was similar to the level of reduction obtained with the use of chemical fungicides, such as Capitan (33.8%) and Ridomil (31.5%) [25]. Other studies have demonstrated that application of *Brevibacillus laterosporus* AMCC100017 can also reduce the PCS disease index by 36.7% under controlled experimental conditions [5]. *Bacillus amyloliquefaciens* Ba01 has also been reported to reduce PCS disease severity by 51.4% in pot assays. Under field conditions, however, the application of *Bacillus amyloliquefaciens* Ba01 only reduced PCS disease severity by 8.8% [9]. Thus far, only a few bioagents have been demonstrated to control PCS under field conditions to a level similar to chemicals, most likely due to the environmental impact on the performance of the bioagents [9].

### 3.2 Modification of Rhizospheric Bacterial Diversity by a Microbial Product Suppresses Plant Disease

**Fig. 2:**
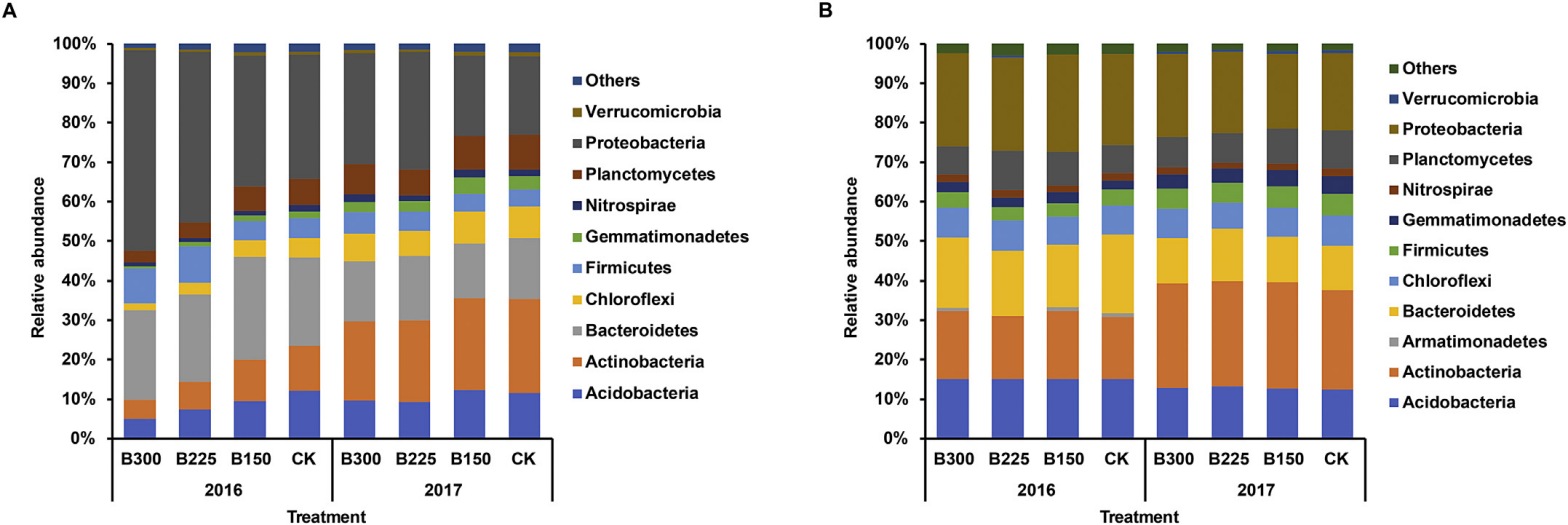
Relative abundance of the most dominant bacterial phyla in potato rhizosphere (A) and bulk soil (B) in 2016 and 2017. CK, B150, B225, and B300 indicate the application rates of 0, 150, 225, and 300 kg·ha^−1^ of a microbial product composed of a consortium of *Bacillus subtilis* and *Trichoderma harzianum.*

PCR amplicons of the 16S rRNA gene were subjected to Illumina sequencing to assess the effect of the microbial product on the composition of the bacterial community in bulk soil and the potato rhizosphere. A total of 867,516 high quality sequences were obtained with an average of 18,073 reads per sample. The obtained sequences were then binned (>97% identity) into 21,682 operational taxonomic units (OTUs). *Proteobacteria* (20–50.7%), *Bacteroidetes* (13.9–26.1%), *Actinobacteria* (4.8–23.9%), *Acidobacteria* (5–12.3%), *Firmicutes* (4.4–9.1%), *Planctomycetes* (3.1–8.7%), *Chloroflexi* (1.8–8%), *Gemmatimonadetes* (0.62–4.2%), *Nitrospirae* (0.9–2.1%), and *Verrucomicrobia* (0.6–0.98%) were the dominant bacterial phyla present in the rhizosphere (Fig. 2A), while *Acidobacteria* (12.4–15.1%), *Actinobacteria* (15.9–27.1%), *Bacteroidetes* (11.3–19.9%), *Chloroflexi* (6.7–7.6%), *Firmicutes* (3.3–5.5%), *Gemmatimonadetes* (2.3–4.5%), *Nitrospirae* (1.5–1.9%), *Planctomycetes* (7.1–9.9%), and *Proteobacteria* (19.1–24.7%) were most prevalent phyla detected in the bulk soil (Fig. 2B). The dominant orders and genera in the rhizosphere and bulk soil are shown in Fig. S1. No significant differences in Chao1 and Shannon indices were detected between treatments in the bulk soil samples in either year (Table 1A). In contrast, α-diversity (Chao1 and Shannon) of the rhizospheric bacterial community decreased in samples where the higher doses of the microbial product had been applied (Table 1B). Chao1 in samples of the B300 and B225 treatments was 35.0% and 22.8% lower, respectively, than in the CK. Shannon index were also 13.3% and 5.6% lower, respectively, than those in the CK in 2016. In 2017, Chao1 in the samples collected from the B300 and B225 treatments was 11.7% and 14.2% lower than in the CK, respectively, while the Shannon index was 2.3% and 2.5% lower than in the CK, respectively (Table 1B). Notably, no significant differences in the Chao1 and Shannon indices of the bacterial community were found between the CK and the B150 treatment in 2017.

**Table 1:** Bacterial α-diversity index for bulk soil (A) and the rhizosphere of potato (B) treated by 0 (CK), 150 (B150), 225 (B225) and 300 (B300) kg·ha^−1^ of microbial product composed of a consortium of *Bacillus subtilis* and *Trichoderma harzianum.*

A					
		B300	B225	B150	CK
2016	Chao1	2970 ± 131.1a	3011 ± 52.6a	2993 ± 178.6a	2934 ± 142.5a
	Shannon	6.60 ± 0.020a	6.59 ± 0.040a	6.59 ± 0.057a	6.57 ± 0.015a
2017	Chao1	2985 ± 123.0a	3143 ± 369.4a	3051 ± 142.3a	2988 ± 94.2a
	Shannon	6.49 ± 0.047a	6.47 ± 0.083a	6.50 ± 0.023a	6.52 ± 0.054a


B					
		B300	B225	B150	CK
2016	Chao1	2055 ± 238.3c	2443 ± 79.0bc	2835 ± 181.9ab	3164 ± 294.9a
	Shannon	5.62 ± 0.071c	6.10 ± 0.040b	6.34 ± 0.080a	6.46 ± 0.044a
2017	Chao1	2879 ± 45.5b	2800 ± 58.1b	3237 ± 69.4a	3262 ± 142.1a
	Shannon	6.36 ± 0.035b	6.35 ± 0.014b	6.50 ± 0.028a	6.51 ± 0.024a

CK, B150, B225 and B300 indicate the application rates of the microbial product were 0, 150, 225 and 300 kg per hectare, respectively. The significance of the differences between the treatments and control was showed by different letters differ significantly (p < 0.05).

**Fig. 3:**
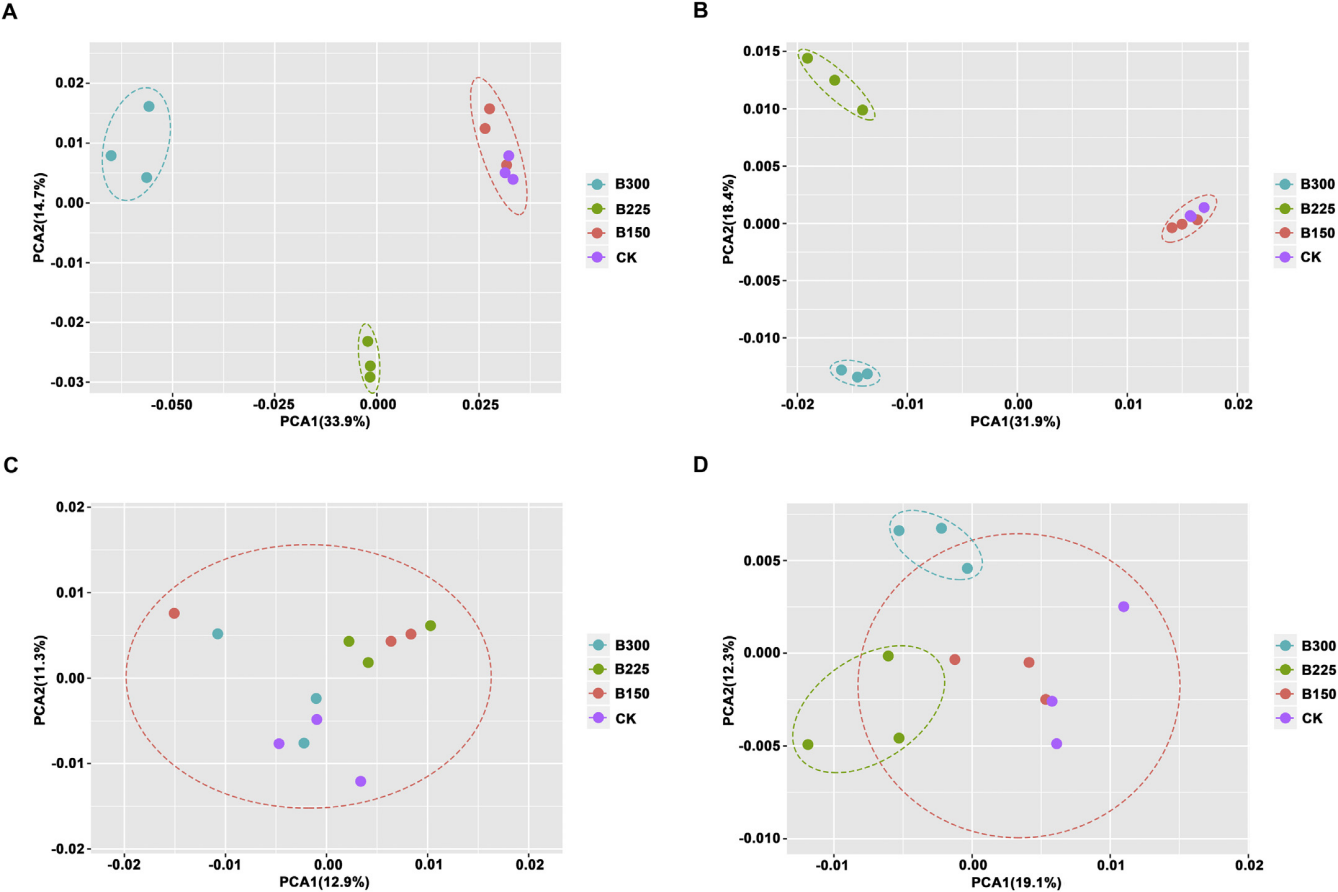
Principle component analysis (PCA) of the bacterial potato rhizosphere community. PCA plot of the bacterial community in the potato rhizosphere in 2016 (A) and 2017 (B), respectively. PCA plot of the bacterial community in bulk soils in 2016 (C) and 2017 (D), respectively. CK, B150, B225, and B300 indicate the application rates of 0, 150, 225 and 300 kg·ha^−1^ of microbial product composed of a consortium of *Bacillus subtilis* and *Trichoderma harzianum.*

PCA analysis was conducted to investigate the effect of the applied microbial product on the bacterial community structure of bulk and potato rhizosphere soils. Results indicated that in both 2016 and 2017, the potato microbial community of samples receiving an application of the microbial consortium were different from the CK, except for the B150 samples. The potato rhizosphere microbial communities also differed between the B300 and B225 treatments (Fig. 3A & B). These results indicate that the high application rate of the microbial product significantly influenced the diversity and structure of the potato rhizosphere bacterial community. The bacterial community in the bulk soil from the CK, B150, B225 and B300 plots sampled prior to the onset of the experiment clustered together in 2016 (Fig. 3C), suggesting that the bacterial community in the bulk soil was similar among the treatment plots prior to the setting up of the experiment. The bacterial community of the bulk soil samples from the B225 and B300 treatment plots tended to separate from samples obtained from the CK or B150 treatment plots in 2017 (Fig. 3D).

In addition, responsive bacterial taxa were identified by partial least squares discriminant analysis (PLS-DA). As indicated in Fig. S4, a clear separation in the rhizosphere community of potato in the different treatments was observed, suggesting that application of higher doses of the microbial product altered the composition of the rhizospheric bacterial communities. The results of PCA and PLS-DA were in general agreement.

The relative abundance of *Proteobacteria* and *Firmicutes* in the potato rhizosphere was higher in the B225 and B300 treatment plots than it was in the B150 and CK treatment plots in both 2016 and 2017 (Fig. 2A). An opposite trend was observed for *Actinobacteria*, *Acidobacteria*, *Chloroflexi*, *Planctomycetes*, *Verrucomicrobia,* and *Gemmatimonadetes* (Fig. 2A). Further analysis revealed that *Pseudomonadales* and *Burkholderiales* were higher in the B225 and B300 treatment plots than other treatment plots in both 2016 and 2017 (Fig. S1A). *Pseudomonadales* in the B300 and B225 plots were 1.9-fold and 1.3-fold higher than that in the CK, while *Burkholderiales* were 1.0-fold and 0.9-fold higher, respectively, than in the CK plots in 2016. In 2017, *Pseudomonadales* in the B300 and B225 plots were 19.3-fold and 22.6-fold higher than in the CK, while the *Burkholderiales* were 0.3-fold and 0.6-fold higher, respectively than in the CK (Fig. S1A). An opposite trend was observed for the *Cytophagales*, *Pirellulales,* and *Solirubrobacterales* (Fig. S1A). At the genus level, *Agrobacterium*, *Achromobacter*, *Bacillus,* and *Pseudomonas* were higher in the B225 and B300 plots, relative to the CK in both 2016 and 2017 (Fig. S1C). The dominant bacterial phyla, orders, and genera exhibited a similar relative abundance in bulk soils obtained from all of the treatment plots in 2016 and 2017 (Fig. 2B, Figs. S1B & S1D).

High throughput sequencing analysis of the 16S rRNA gene indicated that the composition of the rhizospheric bacterial community was different in plots treated with a high-dose of the microbial product, composed of a consortium of *Bacillus subtilis* and *Trichoderma harzianum*, than it was in the potato rhizosphere of non-treated plots or those treated with a low-level (B150) of the microbial product. This result is in agreement with previous reports that soil-borne diseases can be suppressed by the application of organic fertilizers, bioagents, or heat treatments and that the suppression can be attributed to changes in the composition of the rhizospheric microbial community [11,26]. Application of higher doses of the microbial consortium caused greater changes in the rhizospheric bacterial community. Correspondingly, PCS disease was less severe in those treatments, suggesting that the ability to modulate the composition of bulk soil or rhizosphere microbial communities is associated with disease control. In both 2016 and 2017, bacterial α-diversity (Chao1 and Shannon indices) was lower in plots that received a high dose of the microbial product.

The effect of bioagents on the α-diversity of plant-related microbial communities varies. Some studies indicate that application of bioagents can increase the α-diversity of the bacterial rhizosphere community [11,[27], [28], [29]], while another study reported that the application of a rice bran amendment suppressed PCS and resulted in a significant decrease in the α-diversity of the rhizosphere bacterial community [30]. It is possible that a higher bacterial α-diversity does not necessarily lead to a lower incidence of plant disease. Several biotic and abiotic factors, including plant species, soil type, and growth stage may have an effect on the α-diversity of the rhizospheric bacterial community [31,32]. Additionally, while thousands of different OTUs may be simultaneously detected in the rhizosphere, only a fraction of them may be involved in disease suppression. The collective data obtained in the present study suggests, however, that changes in bacterial community composition by the application of a microbial product are associated with suppression of PCS.

### 3.3 The Response of Bacterial Taxa in the Potato Rhizosphere to the Application of Microbial Consortium and Its Association With PCS Suppression and Yield Improvement

**Fig. 4:**
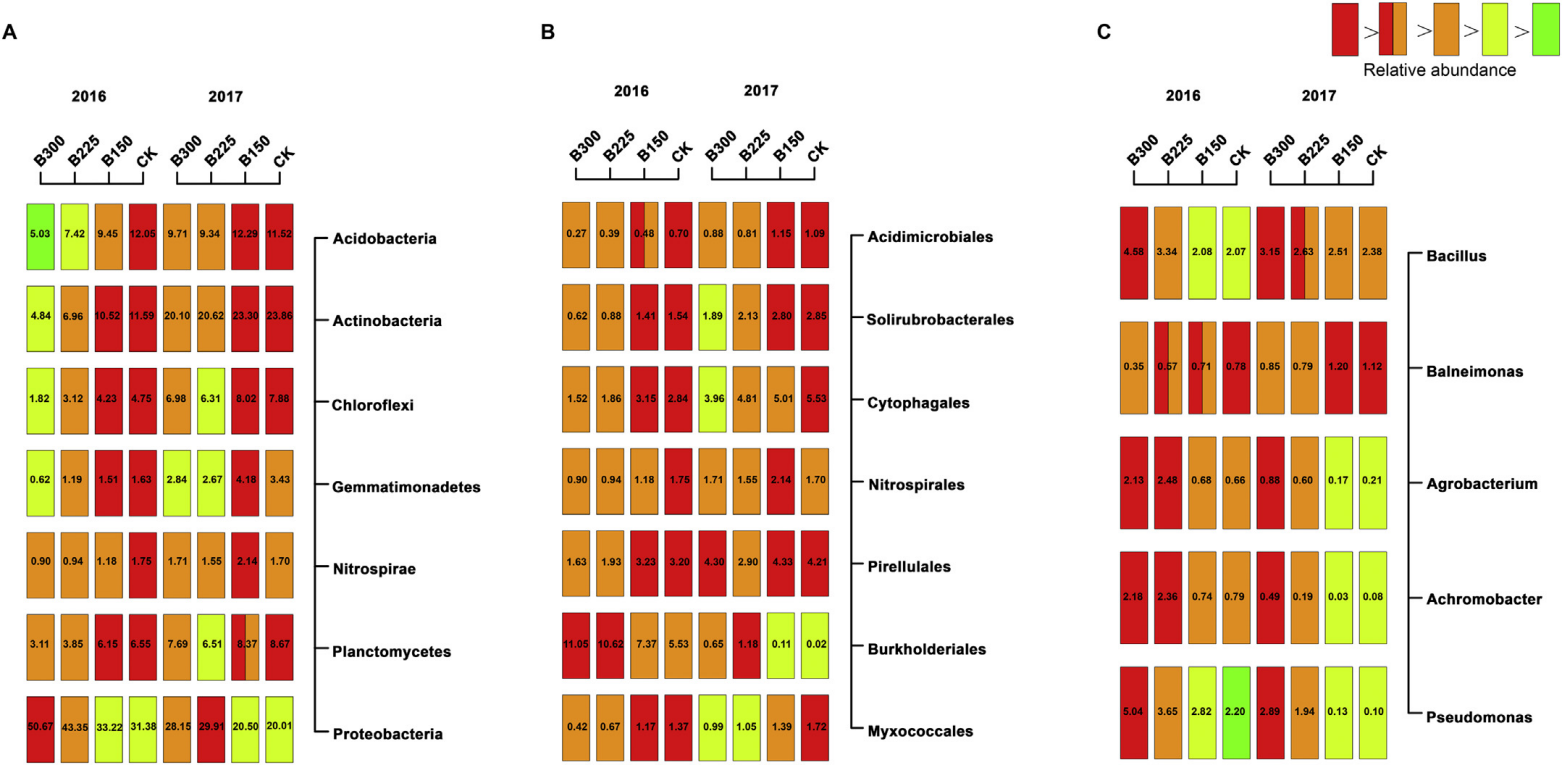
The relative abundance of bacterial phyla (A), orders (B), and genera (C) in the potato rhizosphere in plots treated with different levels of a designated microbial product composed of a consortium of *Bacillus subtilis* and *Trichoderma harzianum.* CK, B150, B225, and B300 indicate the application rates of 0, 150, 225, and 300 kg·ha^−1^ of the designated microbial product. Numbers on the rectangle indicate relative abundance expressed as a percentage. Different colored rectangles indicate a significant difference between treatments (p < 0.05). A rectangle with two colors indicates no significant difference between that treatment and treatments with rectangles of either of the two colors.

**Fig. 5:**
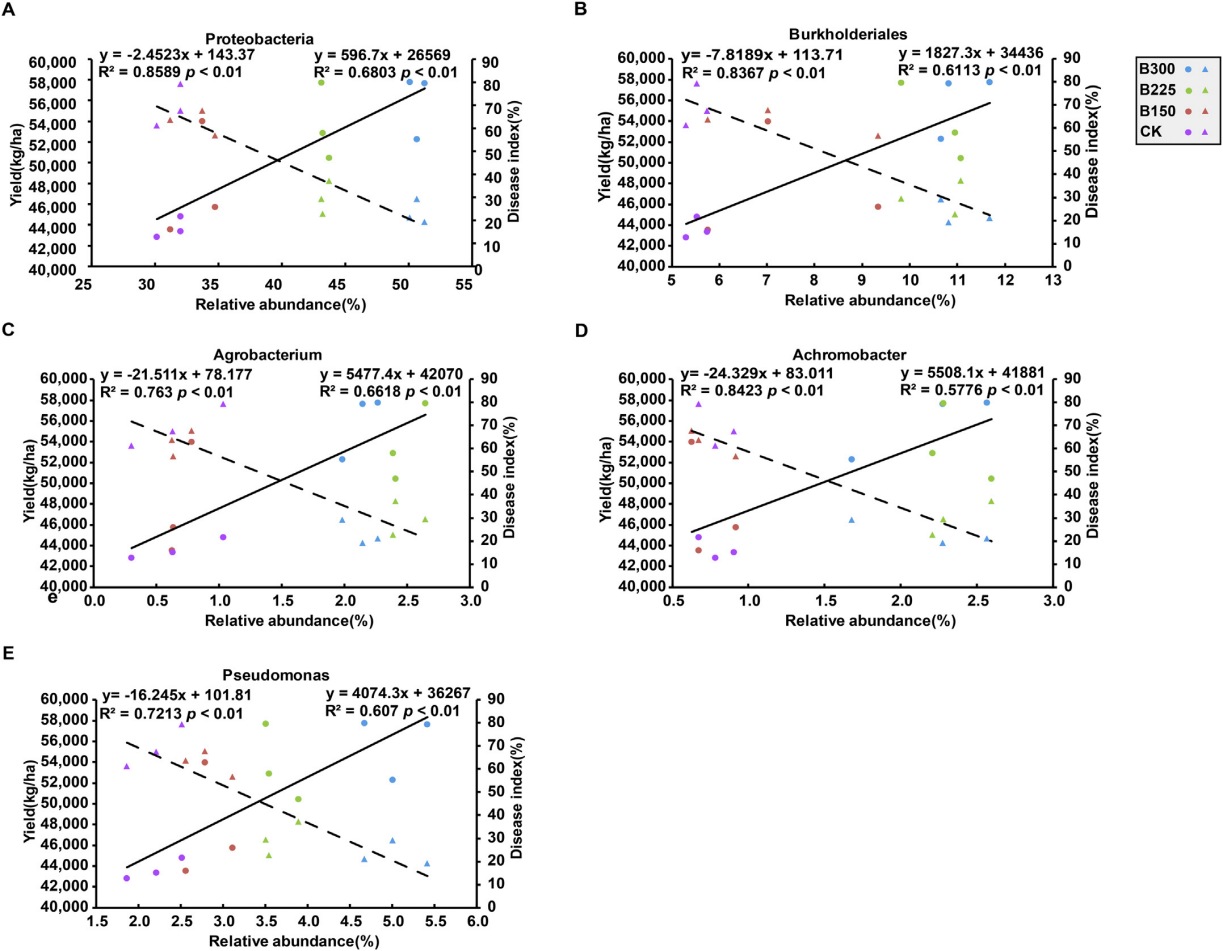
Regression analysis of the relationship between the relative abundances of dominant bacterial phyla (A), orders (B) and genera (C, D, E) in the potato rhizosphere with PCS disease severity or tuber yield in 2016. The relative abundance of phyla, orders and genera were significantly higher in plots treated with 225 (B225) or 300 (B300) kg·ha^−1^ of a microbial product composed of a consortium of *Bacillus subtilis* and *Trichoderma harzianum* than in plots treated with 150 (B150) kg·ha^−1^ of the microbial product or non-treated (CK). The relationship between the relative abundance of each of the designated bacterial taxa in each treatment and tuber yield is indicated by circles; solid lines represent fitted regression lines of the relationship between the relative abundance of each of the designated bacterial taxa and tuber yield. The relationship between the relative abundance of each of the designated bacterial taxa in each treatment and PCS disease index is indicated by triangles; dotted lines represent fitted regression lines of the relationship between the relative abundance of each of the designated bacterial taxa and PCS disease index.

**Fig. 6:**
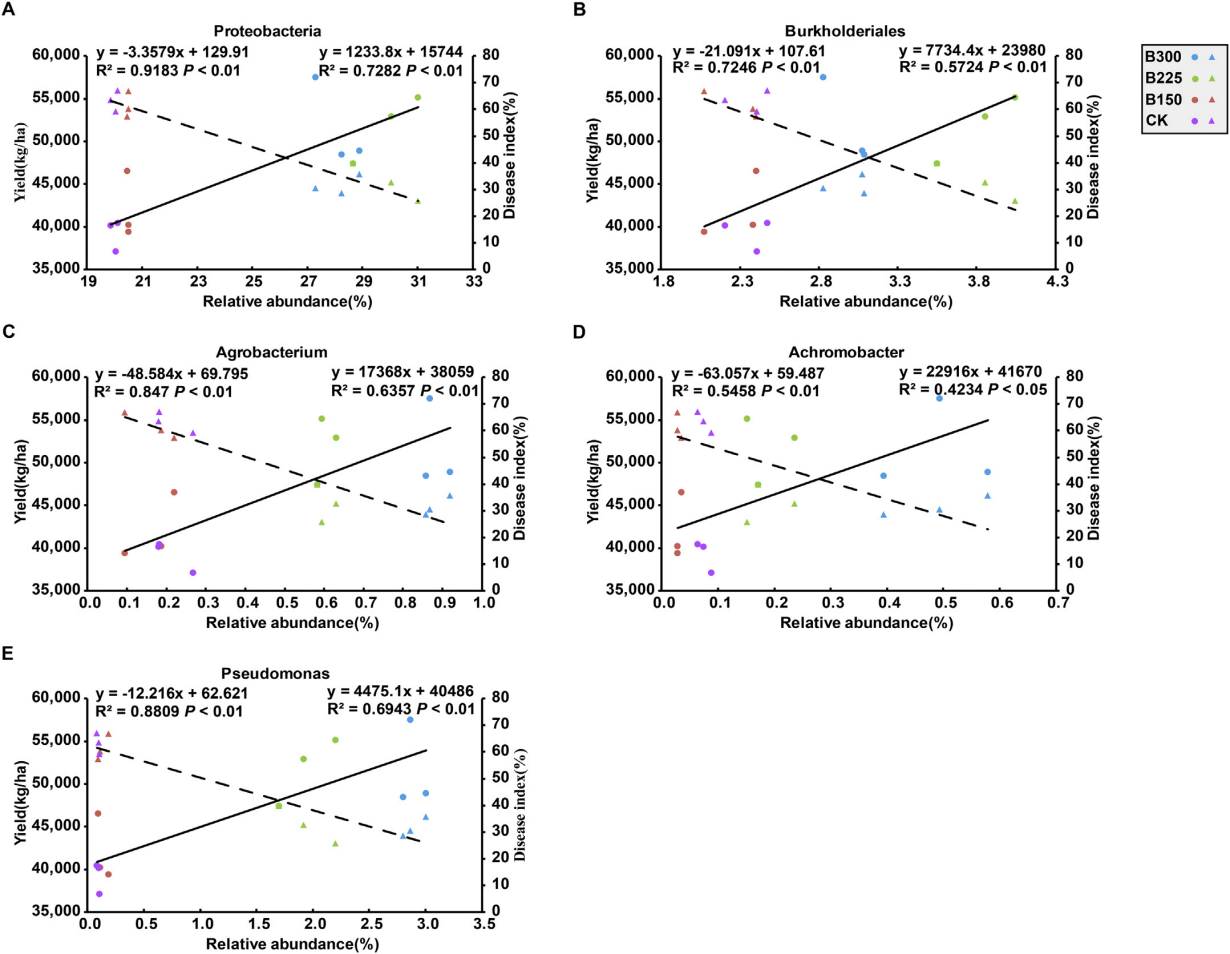
Regression analysis of the relationship between the relative abundances of dominant bacterial phyla (A), orders (B), and genera (C, D, E) in the potato rhizosphere of potato and PCS disease severity and tuber yield in 2017. The relative abundance of phyla, orders and genera were significantly higher in plots treated with 225 (B225) or 300 (B300) kg·ha^−1^ of the microbial product composed of a consortium of *Bacillus subtilis* and *Trichoderma harzianum* than in plots treated with 150 (B150) kg·ha^−1^ of the microbial product or non-treated (CK). The relationship between the relative abundance of each of the designated bacterial taxa in each treatment and tuber yield is indicated by circles; solid lines represent fitted regression lines of the relationship between the relative abundance of each of the designated bacterial taxa and tuber yield. The relationship between the relative abundance of each of the designated bacterial taxa in each treatment and PCS disease index is indicated by triangles; dotted lines represent fitted regression lines of the relationship between the relative abundance of each of the designated bacterial taxa and PCS disease index.

Multiple regression analyses were conducted to determine the relationship between the composition of the bacterial potato rhizosphere community and potato health and tuber yield. In both years, the relative abundance of *Proteobacteria* in the rhizosphere of plants in plots that received the B225 or B300 treatment was significantly higher, while the *Acidobacteria*, *Actinobacteria*, *Chloroflexi*, and *Gemmatimonadetes* were significantly lower than in the rhizosphere of plants in the B150 and CK treatment plots (Fig. 4A). The relative abundance of *Proteobacteria* was negatively correlated with PCS disease severity (R^2^ = 0.86 and 0.92 in 2016 and 2017, respectively, p < 0.01) and positively correlated with tuber yield (R^2^ = 0.68 and 0.73 in 2016 and 2017, respectively, p < 0.01) (Fig. 5 & Fig. 6). At the order level, the relative abundance of the *Burkholderiales* was significantly higher in both years in the plant rhizosphere in plots receiving the higher doses of the microbial product, while the *Myxococcales* and *Solirubrobacterales* were significantly lower, relative to the B150 and CK treatment plots (Fig. 4B). A positive relationship between tuber yield and the relative abundance of *Burkholderiales* was observed in both years (R^2^ = 0.61 in 2016; R^2^ = 0.57 in 2017; p < 0.01), and a significant negative relationship was observed between disease severity and relative abundance in both years (R^2^ = 0.84 in 2016; R^2^ = 0.72; p < 0.01) (Figs. 5 & 6). Compared to the CK and B150 treatments, the relative abundance of the genera, *Agrobacterium*, *Achromobacter,* and *Pseudomonas* was significantly higher in the rhizosphere of potato plants in the B225 and B300 treatment plots than it was in the CK and B150 treatment plots in both 2016 and 2017 (Fig. 4C). PCS disease severity was significantly negatively correlated with the relative abundance of *Agrobacterium*, *Achromobacter*, and *Pseudomonas* (R^2^ = 0.76, 0.84, and 0.72 in 2016, respectively; R^2^ = 0.85, 0.55, and 0.88 in 2017, respectively; p < 0.01). Tuber yield exhibited a significant positive relationship with the relative abundance of *Agrobacterium*, *Achromobacter*, and *Pseudomonas* (R^2^ = 0.66, 0.58, and 0.61 in 2016, respectively; R^2^ = 0.64, 0.42, and 0.69 in 2017, respectively; p < 0.01) (Figs. 5 & 6).

In contrast, the relative abundance of *Acidobacteria*, *Actinobacteria*, *Chloroflexi*, and *Gemmatimonadetes* were positively correlated with PCS disease severity (R^2^ = 0.79, 0.85, 0.80, and 0.70 in 2016, respectively; R^2^ = 0.89, 0.74, 0.82, and 0.71 in 2017, respectively; p < 0.01) and negatively correlated with tuber yield (R^2^ = 0.69, 0.65, 0.59, and 0.50 in 2016, respectively; R^2^ = 0.68, 0.54, 0.62, and 0.52 in 2017, respectively; p < 0.01) (Figs. S2 & S3). At the order level, the relative abundance of *Myxococcales* and *Solirubrobacterales* were positively correlated with PCS disease severity (R^2^ = 0.95 and 0.74 in 2016, respectively; 0.87 and 0.83 in 2017, respectively; p < 0.01) and negatively correlated with tuber yield (R^2^ = 0.50 and 0.62 in 2016, respectively; 0.75 and 0.66 in 2017, respectively; p < 0.01) (Figs. S2 & S3).

The importance of soil or plant microbiomes in plant health has been increasingly recognized [[33], [34], [35]]. In the present study, an increased relative abundance of *Proteobacteria,* such as *Burkholderiales*, *Pseudomonas*, *Achromobacter*, and *Agrobacterium* was observed in the potato rhizosphere at the early stage of tuber formation and development. Stimulation of these taxonomic groups in the rhizospheric microbial community by the application of a microbial product, composed of a consortium of *Bacillus subtilis* and *Trichoderma harzianum*, was also observed in other plants such as apple [27,36]. These taxonomic groups are known to harbor many beneficial microorganisms that can promote plant growth, and suppress soil-borne diseases, such as PCS [36,37]. The relative abundance of *Agrobacterium* spp. has been reported to be associated with an increase in soil fertility and crop yield [36]. A correlation analysis indicated that the relative abundance of these taxonomic groups was negatively correlated with PCS severity (PCS disease index) and positively correlated with tuber yield, suggesting that members of this genus may contribute to the suppression of PCS and yield improvement. Bacterial taxa in the *Burkholderiales* have been recently reported to be plant growth-promoting rhizobacteria (PGPR) that stimulate plant growth [38]. Bioagents in the genus, *Pseudomonas*, also have the potential to reduce the incidence of PCS under field conditions [16,30]. Collectively, our results indicate that application of the designated microbial product contributes to an increase in the relative abundance of beneficial bacteria in the rhizosphere of potato. The specific mechanisms associated with PCS suppression and the recruitment of beneficial microorganism by the microbial product, however, remain to be explored.

### 3.4 Microbial Product Amendments and Soil Microbiome Management

Soils represent a rich reservoir for identifying and obtaining a variety of microorganisms that improve plant health and/or nutrient acquisition [33]. Harnessing the soil microbiome was suggested to be an important strategy in developing sustainable agriculture practices [39,40], especially when the use of high levels of agrochemicals increase crop productivity but cause severe disruption to beneficial organisms and create environmental pollution. Different management approaches, such as the application of organic fertilizer, frequently cause a shift in the structure of soil microbial communities by stimulating the activity of indigenous microorganisms. The effect of organic fertilizer on the soil microbial community rapidly decreases with time, thus it is unlikely that it would affect the soil microbial community after the first year of application. Our results, however, suggest that the application of 225 kg·ha^−1^ or 300 kg·ha^−1^ of a microbial product, composed of a consortium of *Bacillus subtilis* and *Trichoderma harzianum*, altered the composition of the soil bacterial community, which could be further influenced by organic fertilizers, plant root exudates, or crop debris [13,30,41]. In the current study, a significant decrease in potato tuber disease (PCS) and a significant increase in tuber yield was observed in plots treated with either 225 kg·ha^−1^ or 300 kg·ha^−1^ of the microbial consoritum. We suggest that these benefits were due to changes in the soil and rhizosphere microbial community, and the interactions of the members within that community, that were induced and driven by the application of the designated microbial product.

## 4 Conclusions

The application of a microbial product, composed of *Bacillus subtilis* strain znjdf1 *and Trichoderma harzianum* strain znlkhc1, suppressed potato common scab disease and increased tuber yield by fostering the recruitment of beneficial bacteria in the potato rhizosphere.

## Conflicts of Interest

The authors declare no conflict of interest.

## Appendix A Supplementary data

Supplementary material Image 1
